# Phylogenetic exploration of hantaviruses in paraguay reveals reassortment and host switching in South America

**DOI:** 10.1186/1743-422X-8-399

**Published:** 2011-08-12

**Authors:** Yong-Kyu Chu, Robert D Owen, Colleen B Jonsson

**Affiliations:** 1 Center for Predictive Medicine for Biodefense and Emerging Infectious Diseases, Louisville, KY 40222 USA; 2 Department of Biological Sciences, Texas Tech University, Lubbock, TX 74909-3131 USA; 3 Department of Microbiology and Immunology, University of Louisville, KY, 40222 USA; 4 Martín Barrios 2230 c/Pizarro, Barrio Republicano, Asunción, Paraguay

**Keywords:** Hantavirus, reassortment, host switching, zoonotic pathogens, ecology, emerging pathogens, phylogenetics, *Akodon, Oligoryzomys*

## Abstract

**Background:**

Longitudinal mark-recapture studies of rodents in two sites in the Mbaracayú Biosphere Reserve in the Interior Atlantic Forest of eastern Paraguay have revealed a complex and intriguing pattern of hantaviruses harbored by rodents in this area. Full-length sequencing and phylogenetic analyses were conducted for several rodents from *Akodon montensis *and *Oligoryzomys fornesi*. The phylogenetic relationships of these viruses were analyzed in the context of hantaviruses in South America with published S- and M-segment sequences.

**Findings:**

Phylogenetic analyses of hantaviruses identified in the Mbaracayú Biosphere Reserve in Paraguay revealed Jabora and Juquitiba viruses are harbored by *Akodon montensis *and *Oligoryzomys fornesi*, respectively. These analyses revealed that in general the constituents of the major subclade for the S- and M-segments differ for the South American hantaviruses. Further, the two major groups within subclade C for the M-segment reflect in general the lethality associated with the viruses within each group.

**Conclusions:**

Phylogenetic studies of Jabora and Juquitiba viruses and other Paraguayan viruses in the context of American hantaviruses revealed reassortment and host-switching in the evolution of South American hantaviruses.

## Findings

Numerous South American hantaviruses can cause hantavirus pulmonary syndrome (HPS) [1]. These viruses are harbored by rodents and studies suggest that each virus has coevolved with its unique rodent reservoir host, which allows viral persistence in the reservoir [2]. Our studies in the Mbaracayú Biosphere Reserve in the Interior Atlantic Forest of eastern Paraguay have revealed a complex and intriguing pattern of hantaviruses harbored by rodents in this area [3]. In Paraguay, Laguna Negra (LAN) virus in *Calomys laucha *was identified as the etiologic agent following an outbreak of HPS in western Paraguay [4].

**Figure 1:**
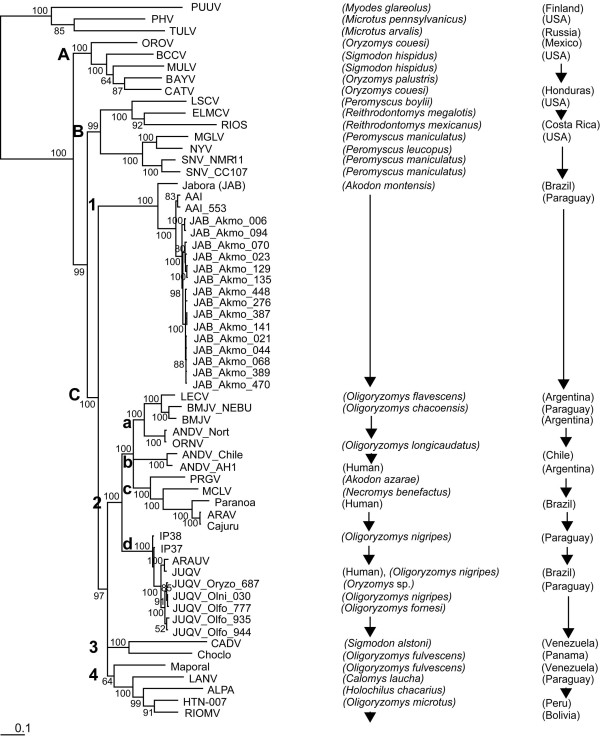
**Bayesian phylogenetic analysis of full length S-segment open reading frame nucleotide sequences of hantaviruses in Paraguay in the context of the Americas**. Abbreviations: *Akodon montensis *is Akmo; *Oligoryzomys fornesi *is Olfo.; Clades are grouped A, B, C; Viral strains abbreviations include Puumula (PUU), Prospect Hill (PH), Tula (TUL), Oro (ORO), Black Creek Canal (BCC), Muleshoe (MUL), Bayou (BAY), Catacamas (CAT), Limestone Canyon (LSC), El Moro Canyon (ELMC), Rio Segunda (RIOS), MGLV, New York (NY), Sin number (SN), Jabora virus (JAB), Ape aime (AAI), Bermejo (BML), (ORN), Andes (AND), Pergamino (PERG), Itapua (IP), Araraquara (ARA), Juquitiba (JUQ), Caño Delgadito (CAD), Choclo, Laguna Negra (LAN), Rio Mamoré (RIOM), Maporal.

**Figure 2:**
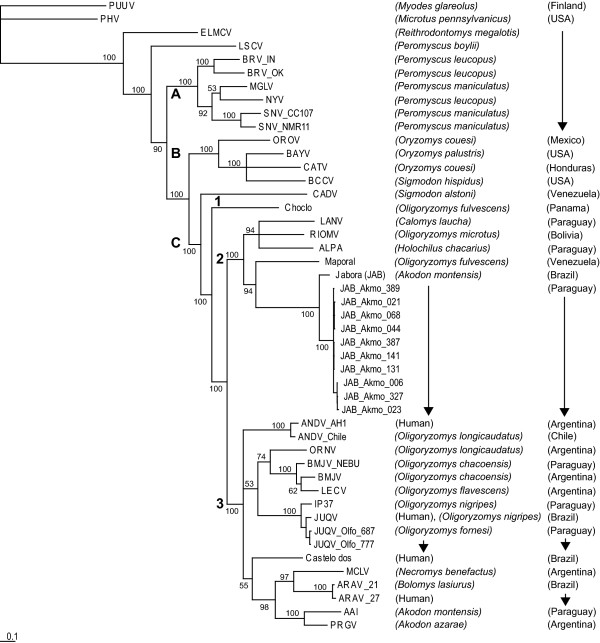
**Bayesian phylogenetic analysis of full length M-segment open reading frame nucleotide sequences of hantaviruses in Paraguay in the context of the Americas**. Abbreviations: *Akodon montensis*is Akmo; *Oligoryzomys fornesi*is Olfo.; Clades are grouped A, B, C; Viral strains abbreviations include Puumula (PUU), Prospect Hill (PH), Tula (TUL), Oro (ORO), Black Creek Canal (BCC), Muleshoe (MUL), Bayou (BAY), Catacamas (CAT), Limestone Canyon (LSC), El Moro Canyon (ELMC), Rio Segunda (RIOS), MGLV, New York (NY), Sin number (SN), Jabora virus (JAB), Ape aime (AAI), Bermejo (BML), (ORN), Andes (AND), Pergamino (PERG), Itapua (IP), Araraquara (ARA), Juquitiba (JUQ), Caño Delgadito (CAD), Choclo, Laguna Negra (LAN), Rio Mamoré (RIOM), Maporal.

We have conducted longitudinal mark-recapture studies of rodents in two sites in the Reserve (Jejui Mi and Horqueta Mi), and two outside (Rama III and Britez Cue) [3]. We have analyzed rodent population dynamics and hantavirus seroprevalence in this subtropical region [5] and reported on sympatry of hantaviruses in *Akodon montensis *and *Oligoryzomys fornesi *[3]. Full-length sequencing and phylogenetic analyses from several rodents from each species support these rodents as reservoirs of genotypes of Jabora virus (JABV) and Juquitiba virus (JUQV), respectively (Figure 1 and 2). JUQV is associated with HPS in cases in Brazil and in northeastern Argentina [6,7], while JABV-like viruses are not [8].

We have made Baysian phylogenetic analyses of these and published S and M sequences that have at least one Kb of sequence from American hantaviruses (Figure 1 and 2). In Figure 1 and 2, for the JABV and JUQV identified in the Reserve, the genotype is indicated as the viral strain followed by the rodent reservoir and identification number. The phylogenetic relationships of subclade C reveal several features in the evolution of hantaviruses in Paraguay and in South America when comparisons are made between S- and M-segments. First, these phylogenetic analyses revealed that in general the constituents of the subclade C for the S- and M-segments differ for the South American hantaviruses. For the S-segment, the phylogenetic tree for subclade C shows four subgroups: C1-JAB strains (Brazil, Paraguay), and a strain (AAI) from Itapúa, Paraguay; C2- Andes (AND), Araraquara (ARA), and Juquitiba (JUQ) viruses (Argentina, Brazil, Chile, Paraguay); C3- Caño Delgadito (CAD), and Choclo viruses (Venezula, Panama); and, C4- LAN, Rio Mamoré (RIOM), Maporal viruses (Bolivia, Paraguay, Peru, Venezula). All the subclade C groups show strong bootstrap values except for C4, which is due to the sequence of Maporal virus. For the M-segment, the phylogenetic tree for subclade C shows three subgroups: C1- Choclo; C2-LAN, RIOM, JAB viruses; and, C3- AND, ARA, JUQ viruses. Notably, the M-segment analyses improved the low bootstrap value for the group with Maporal virus through creation of an independent subgroup (compare M-segment C2 and S-segment C4).

Secondly, we noted two major groups within subclade C for the M-segment that reflect in general the lethality associated with the viruses within each group. Most of the viruses in subclade C2 show a mortality in HPS cases of ~5-10%. In contrast, many of the viruses in subclade C3 show a very high mortality of 40-50%. The S-segment phylogenetic tree further subdivides the M-segment C2 subclade into the JABV group (S-segment C1) and the LANV/RIOM group (S-segment C4). At present, we have no information on whether viruses of the JABV group cause HPS. The amino acid homologies of representatives of these viruses also break into two major groups based on low or high severity of HPS (Table 1).

**Table 1:** Amino acid sequence similarity of S and M segment among hantaviruses identified in Paraguay and nearby countries

	LAN	RIO M	ALPA	JAB	JAB Akmo 006	AAI	AND	BMJ-NEB	IP37	JUQV Olfo 777	JUQ	PRG	ARA
**LAN**	-	96	94	92	90	94	94	94	94	94	94	94	NA

**RIOM**	93	--	94	92	90	92	94	94	94	94	94	93	NA

**ALPA**	92	96	--	94	94	95	94	94	96	96	96	95	NA

**JAB**	85	89	88	--	97	94	93	93	94	94	95	95	NA

**JAB Akmo 006**	86	88	88	99	--	94	93	93	94	94	95	95	NA

**AAI**	88	90	90	99	99	-	95	95	96	96	96	96	NA

**AND**	90	90	89	86	86	88	-	98	98	96	96	95	NA

**BMJ-NEB**	89	90	88	86	85	88	98	-	98	97	98	98	NA

**IP37**	90	90	89	86	86	88	96	95	-	99	99	98	NA

**JUQ Olfo 777**	89	88	87	86	86	87	96	94	100	-	99	99	NA

**JUQ**	90	90	89	86	85	88	96	95	100	100	-	97	NA

**PRG**	90	90	89	85	84	87	96	95	93	92	93	-	NA

**ARA**	91	91	90	90	89	90	96	94	94	94	94	96	-

Above -: M segment amino acid sequence similarity

Below -: S segment amino acid sequence similarity

Our analyses further revealed a reassortment of AAIV (harbored by *A. montensis*), identified in Itapúa, Paraguay [9], that falls in subclade C3 in the M-segment. AAIV shows a strong relationship with Pergamino virus (PRGV), originally identified in Argentina in *A. azarae*. However, for the S-segment, AAIV shows a strong relationship with JABV. In agreement with *in vitro *published reassortant studies of ANDV [10], the AAIV genotype was a reassortment of the S-segment of the JABV-like viral genotypes and the M-segment of the AND-like viral genotypes. The direction of the reassortment would suggest spillover of the AND-like viral genotype into an *A. montensis*, which would have necessarily harbored a JABV at that time. Even more intriguing is the grouping of JABV in the S- and M-Segment analyses. The JABV strain clearly shows a strong relationship in the M-segment C-2 subclade with the LAN and RIOM viral strains. However, the S-segment analyses reveal that the JAB and AAI are more closely related, and remarkably, these strains do not cluster with any of the other subclades, C2-4.

Finally, the phylogenetic trees do not support strong associations of any host phyletic groups within any subclade. The lack of association with the rodent groups argues that unlike hantaviruses in Europe and Asia [2], hantaviruses do not show the level of coevolution with their hosts in South America and hence these viruses have great potential for host switching and adaptation. Certainly, the recent radiation of the Sigmodontinae in South America [11,12] reflects a more recent introduction of hantaviruses into South America. Molecular clock analyses suggest that hantaviruses were introduced approximately 800 years ago [13].

In summary, future studies that integrate large scale phylogeographic mapping coupled with local molecular phylogenetic analyses of rodent-hantavirus relationships in the Americas have great potential to address important questions in the ecology of zoonotic pathogens such as the molecular events that lead to transfer and adaptation to a new host. In South America, events that lead to transmission, host switching, recombination, reassortment and post-transfer adaptation have not been addressed. These questions are critical to interpretation of ecological trends in the emergence and spread of zoonotic diseases, causes of outbreaks, and importantly, guidelines for control and prevention of disease.

## Competing interests

The authors declare that they have no competing interests.

## Authors' contributions

YKC participated in the cloning, sequencing, sequence alignment and phylogenetic analyses. YKC, RDO and CBJ participated in the design of the study. YKO, RDO and CBJ conceived of the study, and participated in its design and coordination and helped to draft the manuscript. All authors read and approved the final manuscript.
